# MicroRNA-15b target Sall4 and diminish in vitro UCB-derived HSCs expansion

**DOI:** 10.17179/excli2014-687

**Published:** 2015-05-08

**Authors:** Mahshid Akhavan Rahnama, Ali Akbar Movassaghpour, Masoud Soleimani, Amir Atashi, Azadeh Anbarlou, Karim Shams Asenjan

**Affiliations:** 1 Hematology & Oncology Research Center, Tabriz University of Medical Sciences, Tabriz, Iran; 2 Department of Hematology, School of Medical Sciences, Tarbiat Modares University, Tehran, Iran; 3 Blood Transfusion Research Center, High Institute for Research and Education in Transfusion Medicine, Tabriz, Iran

**Keywords:** HSCs, expansion, Sall4, miR-15b, miR-219-5p

## Abstract

Hematopoietic Stem Cells (HSCs) are cells that have the ability to self-renewal and differentiate into all of hematopoietic lineages. The lack of donors and unavailable efficient protocols for ex vivo expansion of HSCs, are obstacles in successful cell therapies. MicroRNAs (also refer as miRNAs or miRs) have significant roles in hematopoiesis; they can effect on HSCs expansion, maintaining undifferentiated state, self-renewal and differentiation. Recently attentions have been given to these small regulatory molecules to utilize them in order to expand HSCs. Using bioinformatics analysis we identified Sall4 as putative target of miR-15b and miR-219-5p. Relative expression levels of miRNAs and Sall4 were evaluated by qRT-PCR. Here we show 247-fold and 4.2-fold increasing Sall4 expression level compared to control group in CD34^+^ cells nucleofected by anti-miR-15b and anti-miR-219-5p, respectively. These data showed that anti-miR-15b can promote clonogenic capacity of HSCs and also we found that miR-15b alone was able to increase the number of CD34^+^HSCs in vitro by more than 2 fold by targeting Sall4. Moreover, level of CD34 marker in HSCs nucleofected by anti-miR-15b increased more than 50 %. Our analysis showed no statistically difference in mRNA level of Sall4 after nucleofection of anti-miR-219-5p. Sall4 is a factor capable of enhancing HSC expansion significantly. We demonstrated that inhibition of miR-15b can enhance ex vivo expansion of UCB-derived HSCs and also expression of Sall4 allowed expansion and preserve self- renewal of CD34^+^ HSCs.

## Introduction

HSCs are multipotent stem cells which reside in specialized microenvironments (niches) in the bone marrow (BM) and defined by two properties: self-renewal capacity and multilineage repopulation ability (Metcalf, 2007[15]). HSCs are forefront of clinical application of stem cells therapy. There are two major problems in HSCs transplantation; low accessibility of suitable human leukocyte antigen (HLA)-matched donors; and the small number stem cells (umbilical cord blood (UCB)) (Csaszar et al., 2012[7]). Isolation HSCs from cord blood is a therapeutic method for patients with haematological disorders such as leukemias, lymphomas, solid tumors, anemias (sickle cell anaemia).

The main disadvantage of UCB-derived HSCs in clinical application is the small number of these precious cells in a single unit (Barker, 2007[4]; Metcalf, 2007[15]). To overcome this problem, many studies have been tried to offer the ways that improve the self-renewal potential and ex vivo expansion of HSCs.

 Generally, protocols for ex vivo expansion of HSCs are based on manipulation of external signaling (using cytokine) (Metcalf, 2008[14]) or internal signaling (by gene therapy techniques). While the cytokine based protocols showed some advantages (e.g. improve in vitro proliferation of HSCs), they are frustrating due to induction of HSCs differentiation (Audet et al., 2002[3]). However, protocols that influence intrinsic regulators are more promising. These regulators are included transcription factors, epigenetic mechanisms, miRNAs and long non-coding RNAs (lncRNAs) (Sauvageau et al., 2004[18]; Walasek et al., 2012[22]). 

MiRNAs are non-coding RNAs (~22nt length) that down-regulate target gene expression at the post-transcriptional level by binding to the 3 ′ untranslated region (UTRs) or coding sequence of their corresponding mRNAs (Tay et al., 2008[21]), resulting in decreased levels of the corresponding protein or cleavage of the RNA (Zhang et al., 2012[26]). However, some miRNAs bind to promoter and induce gene expression, a mechanism that called miRNA-mediated RNA activation (Huang et al., 2012[11]). Studies have signified the importance of miRNAs in regulation of various biological processes such as development, proliferation, apoptosis and differentiation (Zimmerman and Wu, 2011[28]). 

One of the important factors involved in self-renewal and maintenance of HSCs multipotency of CD34 stem cells is Sall4 transcription factor (TF) (Aguila et al., 2011[1]). Sall4 is zinc-finger TF belong to spalt-1ike protein family, located on chromosome 20 and expressed in undifferentiated stem cells like normally embryonic stem cells (ESCs) and CD34^+^ HSCs (Tay et al., 2008[21]). Sall4 binds to regulatory regions of ESCs master regulators such as Nanog and Oct-4 and regulates their expression (Wu et al., 2006[23]; Zhang et al., 2006[25]; Tan et al., 2013[20]). This TF is crucial for embryo development and has an important role in preserving of self-renewal in mesenchymal stem cells (MSC) and HSCs. Moreover, expression of Sall4 represses during differentiation with other pluripotency related genes and is going to express pluripotency genes. Recently, Sall4 is known as important factor in expansion process in of HSCs (Zhang et al., 2006[25]; Aguila et al., 2011[1]). 

In current study, by using bioinformatics analysis we identified Sall4 as putative target of miR-15b and miR-219-5p and showed that UCB-derived HSCs bearing two anti-miRs for miR-15b and miR-219-5p exhibit enhanced expansion efficiently in vitro.

## Materials and Methods

### Immunomagnetic separation and cell culture

UCB was prepared from Iranian Blood Transfusion Organization. Mononuclear cells were isolated from UCB by Ficoll-Paque PLUS (GE Healthcare) density gradient centrifugation. The MACS indirect CD34 MicroBead Kit (human-Miltenyi Biotec) was used to separate CD34^+^ HSCs according to the manufacturer's instructions. Afterwards, CD34^+^ HSCs were collected and transferred into serum-free Stemline II expansion medium (Sigma-Aldrich) supplemented with 50 ng/ml TPO, 50 ng/dl SCF, 50 ng/dl Flt-3, 2 mmol/l l-glutamine and 1x antibiotics.

### Flow cytometry

The purity of CD34^+^ HSCs was evaluated by Flow cytometry. Cells were incubated with phycoerythrin (PE)-conjugated anti-CD34 antibodies (Miltenyi Biotec) for 45 min at 4 °C. Mouse IgG1was used as isotype control. Evaluation of CD34^+^ cells also were performed in days 7 and 10. 

### Plasmid preparation and nucleofection

Lenti-miR-Off-GFP-hsa-miR-15b vector (anti-miR-15b), Lenti-miR-Off-GFP-hsa-miR-219-5p vector (anti-miR-219-5p) and pLenti-III-GFP-mir-Off control vector (ABM Inc., Canada) were purchased from ABM Incorporation. Bacterial colonies were grown in LB-ampicillin broth by incubation with shaking at 37 °C overnight. Then plasmids were extracted using the DNA purification kit (DNA-spin, iNtRON Biotechnology, Seoul, Korea).

Nucleofection of CD34^+^ HSCs was performed according to the manufacturer's instructions with the nucleofector machine (Lonza). Cells (8×10^5^) were resuspended in 100 μL of HSCs nucleofection solution (Lonza), and 5 μg of anti hsa-miR-15b, 5 μg of anti miR-219-5b, and 5 μg of control scramble vector (abm Inc., Canada) was added. Samples were transferred into certified cuvettes (Lonza) and transfected by nucleofection with program U-008. Fresh medium (500 μL) was added immediately after transfection to each cuvette, and the cells were plated and incubated at 37 °C for 10 days. After nucleofection, HSCs were stained with 0.4 % Trypan Blue (vital dye) (Sigma-Aldrich) for 1 min at room temperature and the number of live HSCs (unstained) were counted every 48 hours using a hemocytometer.

### Colony forming unit assay

The clonogenic capacity was evaluated before and after expansion by colony forming unit (CFU) assay. Approximately 1500 cells were mixed in 2 ml of methocult H4435 (Stem Cell Technology). Then plates incubated at 37 °C for 14 days. The number of colony CFU was counted after 14 day using an inverted microscope. 

### RNA extraction and qRT-PCR for mRNA

Total RNA was extracted with Trizol (Invitrogen). Reverse transcription was performed using a M-MuLV RT Master Mix kit (CinnaGen Co.). The cDNA were subjected to qRT-PCR using SYBR Premix Ex Taq II (Takara Bio Inc.). β-actin mRNA in each sample was quantified as an endogenous control. The relative expression levels of Sall4 were calculated using the 2^-ΔΔCt^ method. The primers used were: Sall4 (forward 5'- GGCGGAGAGGGCAAATAACT -3', reverse 5'-CACTGGAGCACCCAGCTC-3'), β-actin (forward 5'-TGAAGATCAAGATCATTGCTCCC-3', reverse 5'-AGTCATAGTCCGCCTAGAAGC-3').

### RNA extraction and qRT-PCR for miRNA

Total RNA was extracted with Trizol (Invitrogen). Reverse transcription was performed using a miRNA 1st-Strand cDNA Synthesis Kit (Stratagene; Agilent Technologies, Inc.). The cDNA were subjected to qRT-PCR (EvaGreen-based qRT-PCR) using High-Specificity miRNA qRT-PCR Detection Kit (Stratagene; Agilent Technologies, Inc.). The relative expression levels of miRNAs were normalized to U6 snRNA as an endogenous control and were calculated using the 2^-ΔΔCt^ method. The primers used were: hsa-miR-219-5p (forward 5'-CTGATTGTCCAAACGCAATTCT-3'), hsa-miR-15b (forward 5'-CTAGCAGCACATCATGGTTTACA -3'), U6 (forward 5'-AAATTGGAACGATACAGAGAAG-3').

### Statistical analysis

All measurements were done in triplicate. Statistical analyses were performed by GraphPad Software (GraphPad PRISM V 5.0analytical Software). Data means were compared using one-way ANOVAs. Statistical significance was defined at P < 0.05.

## Results

### Evaluation of efficacy of nucleofection by GFP Vector

**Figure 1:**
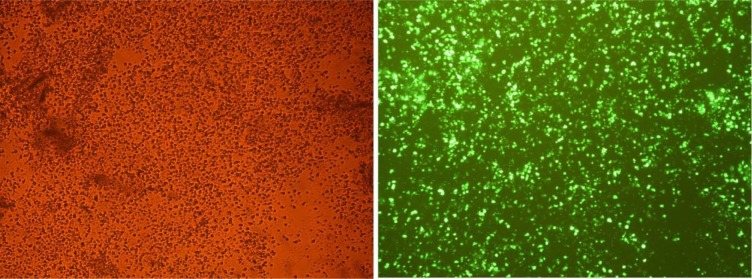
Nucleofection of HSCs. Nucleofection efficiency was 75 % using pmax-GFP vector for the HSCs 48 hours post nucleofection. Magnification, ×200

To further show the role of hsa-miR-15b and hsa-miR-219-5p in HSCs, nucleofection of the HSCs was done by anti-miR-15b, anti-miR-219-5p and control vector. Nucleofection efficiency was evaluated by EclipseTE-2000 Fluorescent microscope (Nikon technologies). More than 75 % of HSCs were nucleofected by pmax-GFP vector after 48 hours post nucleofection (Figure 1(Fig. 1)).

### Effects of anti hsa-miR-15b and anti miR-219-5p on ex vivo expansion of CD34^+^ cells

To clarify, whether down-regulation of miR-15b and miR-219-5p could lead to up-regulation of Sall4 and eventually promote CD34^+^ HSCs expansion, cells were transfected with anti-miR-15b, anti-miR-219-5p and control vector using nucleofection technique. MiR-15b and miR-219-5p was predicted to induce hematopoietic differentiation due to targeting Sall4 in different levels.

**Figure 2:**
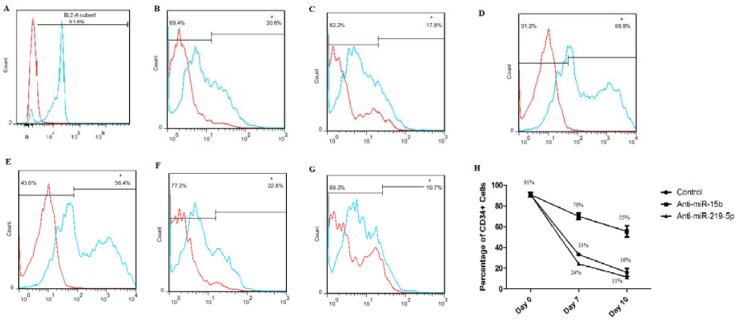
Flow cytometry results. Released data (A-G) is one of 3 repetitions. Purity of CD34^+^ HSCs in Day 0 (A); Percentage of CD34^+^ cells in control vector group in day 7 (B) and day 10 (C); Percentage of CD34^+^ cells in anti-miR-15b group in day 7 (D) and day 10 (E); Percentage of CD34^+^ cells in anti-miR-219-5p group in day 7 (F) and day 10 (G); Graph shows percentage of CD34^+^ cells in days 0, 7 and 10 after nucleofection in 3 repetitions. Values are shown as mean ± SEM (H).

The purity of CD34^+^ cells was evaluated by flow cytometry in days 0, 7 and 10. The percentage of CD34^+^ Cells was nearly 91 % after immunomagnetic separation (Figure 2(Fig. 2)). Then, we compared percentage of CD34^+^ cells to our groups in days 7 and 10. The highest level of CD34 marker belonged to the anti-miR-15b group that was 70 % and 55 % in days 7 and 10, respectively (Figure 2(Fig. 2)).

The percentage of CD34 marker in cells treated by anti-miR-219-5p (24 % and 11 % in days 7 and 10, respectively) was lower than control group in both days (33 % and 16 % in days 7 and 10, respectively) (Figure 2(Fig. 2)).

**Figure 3:**
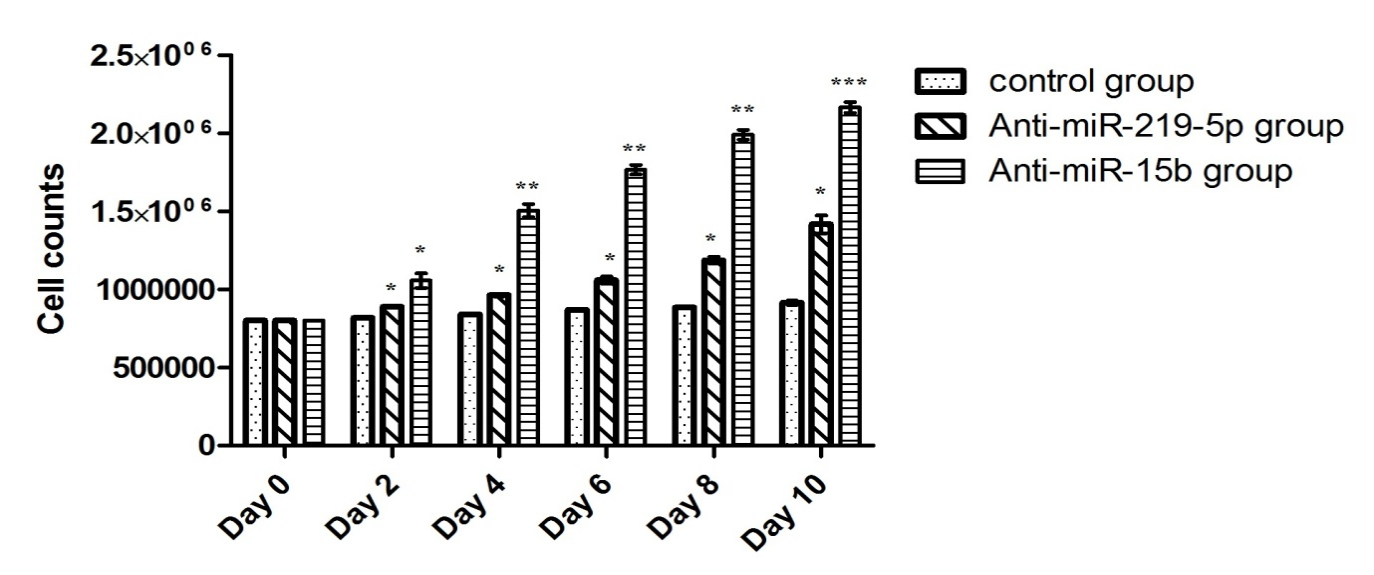
Bar graph shows total cell counts. Total cell counts were determined at every 48 hours. Values are shown as mean ± SEM. Statistically different values of *P < 0.05 and **P < 0.01 and ***P < 0.001 are determined compared with the control.

HSCs viability and total cell counts were determined at every 48 hours by trypan blue exclusion and it was always above 80 %. HSCs nucleofected with anti-miR-15b expanded 2.3 fold (P < 0.001) and anti-miR-219-5p 1.5 fold (P < 0.05) compared to those nucleofected with control vector (Figure 3(Fig. 3)). Based on the results of flow cytometry for expansion marker CD34, in anti-miR-219-5p group, we resulted that this effect was not a result of self-renewal effects on HSCs. Interestingly, inhibition of miR-15b expression with anti-miR redounded Sall4 expression that was concurrent with rises in HSCs expansion.

### Hematopoietic colony formation assay

**Figure 4:**
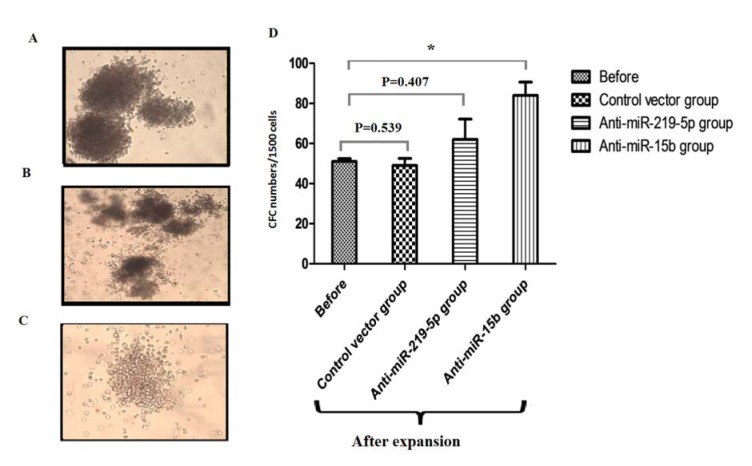
Numbers of colony forming units in different groups after 14 days. (A) Burst-Forming Unit-Erythroid (BFU-E); (B) CFU- Granulocyte, Erythrocyte, Monocyte/macrophage, Megakaryocyte (CFU-GEMM); (C) CFU-Granulocyte-Macrophage (CFU-GM); Magnification, ×100. (D) Bar graph shows the number of CFC in different groups after 14 days; Values are shown as mean ± SEM. Statistically different values of *P < 0.05 and **P < 0.01 and ***P < 0.001 are determined compared to the before group.

Clonogenic capacity of HSCs was evaluated before and after expansion. As shown in Figure 4(Fig. 4), the number of CFCs increased on average 1.6-fold (P= 0.039) in nucleofected HSCs by anti-miRNA-15b.HSCs treated with vector control(P=0.539) and anti-miR-219-5p (P=0.407) showed no statistically significant difference in the clonogenic capacity (Figure 4(Fig. 4)).These data demonstrate that anti-miR-15b can promote clonogenic capacity of HSCs.

### Effects of anti-miR-15b and anti miR-219-5p on Sall4 expression 

As biological significance of miRNA relies on inhibition their gene targets, we analyzed the predicted targets of hsa-miR15b and hsa-miR-219-5p. The analysis was performed by using three algorithms, mirBase, miRanda and TargetScan commonly used to predict human miRNA gene targets. We found that the expression of Sall4 showed an inverse correlation with the expression of hsa-miR-15b and hsa-miR-219-5p in CD34^+^ HSCs. We hypothesized hsa-miR-15b and hsa-miR-219-5p have potential for targeting Sall4. As predicted by mirBase, 3' UTR of Sall4 had complementary sequence for has-miR-15b and has-miR-219-5p seed sequence.

To assert whether hsa-miR-15b and hsa-219-5p efficacy could retrieveSall4expression, anti-miR-15b and anti-miR-219-5p were nucleofected in CD34^+^ HSCs. Expression levels of Sall4 were detected by qRT-PCR.

Real-time PCR showed that anti-miR-15b and anti-miR-219-5p can rectify Sall4 down-regulation, resulted in an increase in cell growth compared with control. 

**Figure 5:**
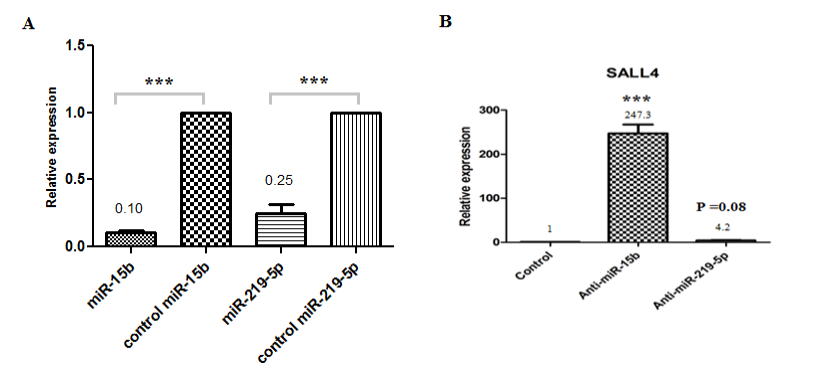
Bar graphs show relative expression levels of miRNAs and Sall4 gene. (A) The relative expression levels of miR-15b in HSCs treated by anti-miR-15bwas 0.1-fold (P < 0.001) and the relative expression levels of miR-219-5p in HSCs treated by anti-miR-219-5p was 0.25-fold (P < 0.001) in comparison to control group; (B) The relative expression levels of Sall4 in HSCs treated by anti-miR-15b and anti-miR-219-5p increase 247.3 (P < 0.001) and 4.2-fold (P=0.08) Sall4 expression in comparison to control group, respectively. Values are shown as mean ± SEM. Statistically different values of *P < 0.05 and **P < 0.01 and ***P < 0.001 are determined compared with the control.

Anti-miR-15b and anti-miR-219-5p increase 247.3 fold (P < 0.001) and 4.2 fold (P=0.08) Sall4 expression in comparison to control group, respectively (Figure 5(Fig. 5)) but the difference in Sall4 expression in anti-miR-219-5p group is not statistically significant. These results indicate that down-regulation of hsa-miR-15b and consequently up regulation of Sall4 lead to in the expansion of primary HSCs. Based on our analyses, miR-15b targets Sall4 mRNA and reduced its expression in HSCs. 

Moreover, qRT-PCR was performed for miRNAs in order to ensure that anti-miRNAs work specifically. Relative expression levels of miR-15b and miR-219-5p in comparison to control group showed in Figure 5A(Fig. 5).

## Discussion

The process of hematopoiesis involves HSCs self-renewal and lineage commitment procedures which are regulated by extrinsic factors (bone marrow microenvironment, cell adhesion molecules, growth factors and cytokines) (Zhu and Emerson, 2002[27]) and intrinsic factors (signalling networks and transcription factors) (Akala and Clarke, 2006[2]).

miRNAs is known as a novel regulatory molecules in hematopoiesis by targeting intrinsic factors involved in hematopoiesis, can effect on HSCs expansion, maintaining self-renewal, differentiation, apoptosis and other biology processes (Bissels et al., 2012[5]; Favreau and Sathyanarayana, 2012[9]). Recently, deregulation of miRNAs has been shown in leukemia that is linked to breakpoint regions associated with chromosome aberrations in leukemias (Zimmerman and Wu, 2011[28]).

Recently, researchers reported miRNAs have roles on the expansion of HSCs. Yang et al. (2013[24]) showed that miR-17 highly expressed on hematopoietic progenitors. The expression of miR-17 is sought on CD34^+^ human HSCs and found that significantly down-regulated while differentiation into monocytes and mature megakaryocytes. Moreover they reported that expansion of cord blood CD34^+^ cell can be partly augmented by osteoblastic miR-17, and ectopic miR-17 can lead to specific expansion of the erythroid lineage through promoting HIF-1a in osteoblasts. Han et al. (2010[10]) suggested that miR-29a may regulate self-renewal in HS/PCs (Hematopoietic stem/progenitor cells). 

Ooi et al. (2010[16]) reported that in the immature hematopoietic system, mir-125b is anti-apoptotic and augments expansion of HCSs. Moreover they showed miR-125b increased HSC engraftment transplants by inhibiting expression of two anti-apoptotic target genes, Bmf (Bcl2 modifying factor) and KLF13 (Krueppel-like factor 13) and they resulted that this effect was a result of self-renewal effects on HSC (not committed progenitors) (Ooi et al., 2010[16]).

In the study on tongue cancer, Sun et al. (2011[19]) showed that one of the target proteins of miR-15b is Bmi-1 which is involved in pluripotency of stem cells. Moreover, studies showed one of the Sall4 downstream genes is Bmi-1 (Jiang et al., 2009[12]).

Choong et al. (2007[6]) were reported that miR-15b has been up regulated in human UCB undergoing erythropoiesis, that is similar to released data by Lu et al. (2005[13]). Based these studies, miR-15b has probably important role in differentiation especially to erythroid lineage that is incompatible with our results.

Rao et al. (2010[17]) demonstrated that over expression of miR-219-5p, decreases proliferation and expansion capacity of brain tumor cells. They reported that miR-219-5p may have important role in development of brain solid tumors (Rao et al., 2010[17]; De Faria et al., 2012[8]). Also, miR-219-5p may have role in oligodendrocyte differentiation by targeting inhibitors of oligodendrocyte differentiation such as Sox6 (De Faria et al., 2012[8]).

Here, we show that miR-15b and miR-219-5p target Sall4 and down-regulation Sall4 occur upon HSCs differentiation. Bioinformatics data suggested that 3' UTR of Sall4 TF has complementary sequences with seed sequences of miR-15b and miR-219-5p. So we asked if these two miRNAs target Sall4, anti-miRNA for these miRs can reverse Sall4 expression and enhance ex vivo expansion of UCB-derived HSCs? Two answer this question, UCB-derived HSCs were nucleofected with anti-miRs against miR-15b and miR-219-5p. Gene expression analysis with qRT-PCR showed an increase in Sall4 expression compared with cells transfected with control vector. 

Flow cytometry for expansion marker, CD34, showed elevation level of CD34^+^ HSCs in cells receive anti-miR-15b (more than 50 %). It has been suggested that this elevation of CD34 level related to express Sall4 gene that result in expansion and maintain self-renewal probably.

Furthermore, colony formation carried out and data showed 1.6-fold increases in colony formation in cells bearing anti-miR-15b. Although number of cells in anti-miR-219-5p group were increased, but these cells could not maintain their self renewality properties.

Altogether, we indicate that inhibition of miR-15b can enhance ex vivo expansion of UCB-derived HSCs. Since, the most drawbacks in transplantation of these cells are leakage of cell numbers, our study purposes an easy way for promoting in vitro HSCs expansion.

Albeit, 3´UTR of miR-219-5p targets Sall4 theoretically, but our analysis showed no difference in mRNA level of Sall4 after nucleofection of anti-miR-219-5p. This data suggests that Sall4 probably is not one of the miR-219-5p targets however, more analysis for confirming this is needed.

## Conclusion

Our studies revealed that miR-15b is expressed in normal HSCs and is up-regulated progressively in mature lineages of HSCs and during hematopoiesis. Transfection of anti-miR-15b in puriﬁed HSCs induces expansion of the HSCs by targeting of Sall4. We report an essential role of Sall4 for HSCs maintenance and the identiﬁcation of miR-15b capable of positively regulating HSCs self-renewal at least in part by increasing expansion levels. 

## Acknowledgements

This research was supported by a grant from the Hematology & Oncology Research Center, Tabriz University of Medical Sciences, Tabriz, Iran. Also, we thank Stem Cell Research Center (BonYakhteh) for supporting technical assistance.

## Conflict of interest

All authors declare no conflict of interest.
